# A dynamic history of admixture from Mediterranean and Carpathian glacial refugia drives genomic diversity in the bank vole

**DOI:** 10.1002/ece3.7652

**Published:** 2021-05-25

**Authors:** Michaela Horníková, Silvia Marková, Hayley C. Lanier, Jeremy B. Searle, Petr Kotlík

**Affiliations:** ^1^ Laboratory of Molecular Ecology Institute of Animal Physiology and Genetics of the Czech Academy of Sciences Liběchov Czech Republic; ^2^ Department of Zoology, Faculty of Science Charles University Prague Czech Republic; ^3^ Department of Biology, Program in Ecology & Evolutionary Biology University of Oklahoma Norman OK USA; ^4^ Sam Noble Museum University of Oklahoma Norman OK USA; ^5^ Department of Ecology and Evolutionary Biology Cornell University Ithaca NY USA

**Keywords:** admixture, genotyping‐by‐sequencing, *Myodes glareolus*, postglacial colonization, SNP

## Abstract

Understanding the historical contributions of differing glacial refugia is key to evaluating the roles of microevolutionary forces, such as isolation, introgression, and selection in shaping genomic diversity in present‐day populations. In Europe, where both Mediterranean and extra‐Mediterranean (e.g., Carpathian) refugia of the bank vole (*Clethrionomys* *glareolus*) have been identified, mtDNA indicates that extra‐Mediterranean refugia were the main source of colonization across the species range, while Mediterranean peninsulas harbor isolated, endemic lineages. Here, we critically evaluate this hypothesis using previously generated genomic data (>6,000 SNPs) for over 800 voles, focusing on genomic contributions to bank voles in central Europe, a key geographic area in considering range‐wide colonization. The results provide clear evidence that both extra‐Mediterranean (Carpathian) and Mediterranean (Spanish, Calabrian, and Balkan) refugia contributed to the ancestry and genomic diversity of bank vole populations across Europe. Few strong barriers to dispersal and frequent admixture events in central Europe have led to a prominent mid‐latitude peak in genomic diversity. Although the genomic contribution of the centrally located Carpathian refugium predominates, populations in different parts of Europe have admixed origins from Mediterranean (28%–47%) and the Carpathian (53%–72%) sources. We suggest that the admixture from Mediterranean refugia may have provisioned adaptive southern alleles to more northern populations, facilitating the end‐glacial spread of the admixed populations and contributing to increased bank vole diversity in central Europe. This study adds critical details to the complex end‐glacial colonization history of this well‐studied organism and underscores the importance of genomic data in phylogeographic interpretation.

## Graphical abstract

Previous mtDNA studies indicated that extra‐Mediterranean refugia provided most of the genetic contributions in the bank vole (*Clethrionomys glareolus*) across its range, while Mediterranean peninsulas harbor isolated, endemic lineages. By analyzing an existing SNP dataset for more than 800 bank voles, we found striking evidence that both extra‐Mediterranean (Carpathian) and Mediterranean (Spanish, Calabrian, and Balkan) refugia contributed to the ancestry and genomic diversity of populations across Europe. We suggest that the admixture from Mediterranean refugia may have facilitated the end‐glacial spread of the admixed populations, contributing to increased bank vole diversity in central Europe.

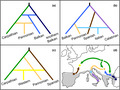

## 1 INTRODUCTION

Understanding the geographic distribution of genetic lineages and the historical partitioning of genetic diversity has been one of the central goals of phylogeography over the last 30 years (Avise et al., 1987). Understanding the role of refugial history, climatic oscillation, and geographic isolation (Hewitt, 2000, 2004) in shaping genetic variation provides insights into the distribution of phenotypic variation (Friis et al., 2018), ecological interactions (Avise et al., 2016), and contemporary connectivity (Lanier et al., 2015). Originally, Mediterranean glacial refugia were considered the main regions for survival of temperate species during unfavorable conditions in the Pleistocene and subsequent sources for end‐glacial colonization of central and northern Europe (Hewitt, 2000; Liepelt et al., 2002; Sommer & Nadachowski, 2006). However, phylogeographic studies have increasingly challenged this view (Pedreschi et al., 2019), indicating that colonization of central and northern Europe may have been substantially driven by extra‐Mediterranean refugia (Bilton et al., 1998; Provan & Bennett, 2008; Schmitt & Varga, 2012). Traditionally, the ascertainment of these patterns has relied on variation in mitochondrial DNA (mtDNA). While the smaller effective population size for the mitochondrial genome is likely to cause mtDNA lineages to track population lineages more closely than an average nuclear marker, it also may fail to capture complex population histories and spatial patterns of genetic diversity (Edwards et al., 2015). Thus, understanding the genetic contribution from differing refugial locations to the recolonization of Europe (Hewitt, 1999) remains an important and largely unrealized goal for most species. Evaluating the relative genomic contributions from differing refugia can provide an important baseline for studies of geographic variation in species’ biological traits (tolerance ranges, adaptive capacity, and plasticity) and how they shape regional distributions.

During the Pleistocene glacial cycles, Mediterranean and extra‐Mediterranean refugia likely experienced different climatic regimes and forced their residents (i.e., separate genetic lineages) to adapt to local conditions (Stewart et al., 2010). Phylogeographic evidence indicates that numerous plant and animal populations isolated in Mediterranean refugia (Iberian, Italian, and, Balkan peninsulas) have accumulated new mutations in situ (Hewitt, 2000; Liepelt et al., 2002). Likewise, unique genetic variation in the vicinity of the Carpathian, Caucasus, Alpine, and Ural Mountains has been used to recognize separate extra‐Mediterranean refugia for a wide variety of species (Deffontaine et al., 2005; Kotlík et al., 2006; Markova et al., 1995; Melnikova et al., 2012; Seddon et al., 2002; Sommer & Zachos, 2009). While much of the variation documented to date involved presumed neutral markers, environmental niche modeling suggests that the niche of some southern populations is associated with warmer climatic conditions compared to the northern lineages (Fletcher et al., 2019; Pearman et al., 2010; Stojak et al., 2019; Wielstra et al., 2012). Gene flow between these different refugial populations may have contributed to adaptation to climatic change and facilitated expansion to the north, following favorable conditions (Kotlík et al., 2014; Strážnická et al., 2018).

Here, we focused on the bank vole *Clethrionomys glareolus* (aka *Myodes glareolus*; Kryštufek et al., 2020), a small mammal species with a broad distribution range in Europe. Bank voles inhabit a large variety of woodland habitats that include Mediterranean mountain and temperate floodplain forests, boreal and subarctic forests, and a diverse range of scrub habitats (Raczyński, 1983). During the last glacial maximum (LGM; 22–17 kyr), bank voles were restricted to multiple allopatric refugia (Filipi et al., 2015) located in the Mediterranean and further north and east, in the vicinity of the Carpathian and Ural Mountains, with the populations emanating from these refugia characterized by distinct mtDNA lineages (Bilton et al., 1998; Deffontaine et al., 2005; Kotlík et al., 2006; Markova et al., 1995). This makes the bank vole an excellent model species for the study of postglacial expansion originating from multiple, disparate refugia. Evidence from environmental niche modeling for the bank vole (Fløjgaard et al., 2009) suggests that suitable Pleistocene climatic conditions existed across central Europe, with species in these extra‐Mediterranean refugia most likely surviving in geographically scattered, low‐density populations (Stewart et al., 2010). For example, suitable habitat for the Carpathian mtDNA lineage (Figure A1 in Appendix S1) was one of the largest during the LGM and mid‐Holocene, compared to the Mediterranean refugial lineages (Escalante et al., 2021). Recent population genomic studies of bank voles (Kotlík et al., 2018; Marková et al., 2020a) demonstrate that the Carpathian refugium contributed to colonization of Britain and Fennoscandia *via* multiple colonization events over land bridges open following the LGM. Although there is now clear evidence that populations deriving from the Carpathian refugia founded end‐glacial colonizations of northern Europe (Marková et al., 2020a), their relative contribution to the ancestry and genomic diversity of the present populations in central Europe remains unclear.

While the latitudinal trend in genomic diversity of bank vole populations indicates the highest genetic diversity in central Europe (Marková et al., 2020a), distinguishing between high diversity as a result of admixture (Petit et al., 2003) *versus* high diversity as a result of refugial size and structure (Bilton et al., 1998; Hewitt, 2000) can provide key insights into the role of different evolutionary forces shaping the species. At the northern apex of bank vole distribution (Britain and Fennoscandia), increased genomic diversity occurs within the areas of secondary contact between populations colonizing newly available areas from different glacial refugia, but it also resulted from multiple waves of colonization from the same refugium occurring at different times (Kotlík et al., 2018; Marková et al., 2020a). In Fennoscandia and Britain, the influx of genes from the continental populations was restricted to certain time periods, before land bridge submersion halted overland colonization. Present admixture in these northern regions is thus currently restricted to that between local populations (Kotlík et al., 2018; Marková et al., 2020a). In contrast, gene flow among populations in central Europe is unlikely to have been interrupted by strong geographic barriers, and may represent an ongoing as well as historical driver of diversity.

Our present study makes use of an existing dataset of single nucleotide polymorphism (SNP) genotype data (Marková et al., 2020b) from an extensive sampling survey of the bank vole across its distribution in Europe (a total of more than 800 individuals from more than 100 localities). In contrast to the study of Marková et al. (2020a), which focused on the drivers of genomic diversity in Fennoscandia, this present paper is focused on populations in the Mediterranean region and in central Europe, which we define broadly as the area north of the Mediterranean region, but excluding the northern apex of the bank vole distribution in Europe, that is, Britain and Fennoscandia (Kotlík et al., 2018; Marková et al., 2020a).

We evaluate how the multiple admixture events between individuals emanating from different refugia shaped the bank vole genetic diversity in central Europe. We test alternative hypotheses about the colonization history of central Europe and its impact on the genetic diversity with a set of nested scenario choice analyses by approximate Bayesian computation *via* random forest (ABC‐RF). Through leveraging the existing extensive SNP dataset, our study of the bank vole provides the first genome‐wide perspective of divergence and admixture between populations emanating from Mediterranean *versus* extra‐Mediterranean (Carpathian) refugia in a widespread European mammal, acting as an important test system.

## 2 MATERIALS AND METHODS

### 2.1 Genomic data

This study involves a new analysis of 6,078 genome‐wide SNP loci (Marková et al., 2020b) generated by Marková et al. (2020a) for 809 bank voles from 103 localities spanning the species distribution range in Europe and representing all major mtDNA lineages (Figure A1 in Appendix S1) previously found in the bank vole (Colangelo et al., 2012; Deffontaine et al., 2005; Filipi et al., 2015; Kotlík et al., 2006; Wójcik et al., 2010). For the bank vole specimen details, see Marková et al. (2020a).

**FIGURE 1:**
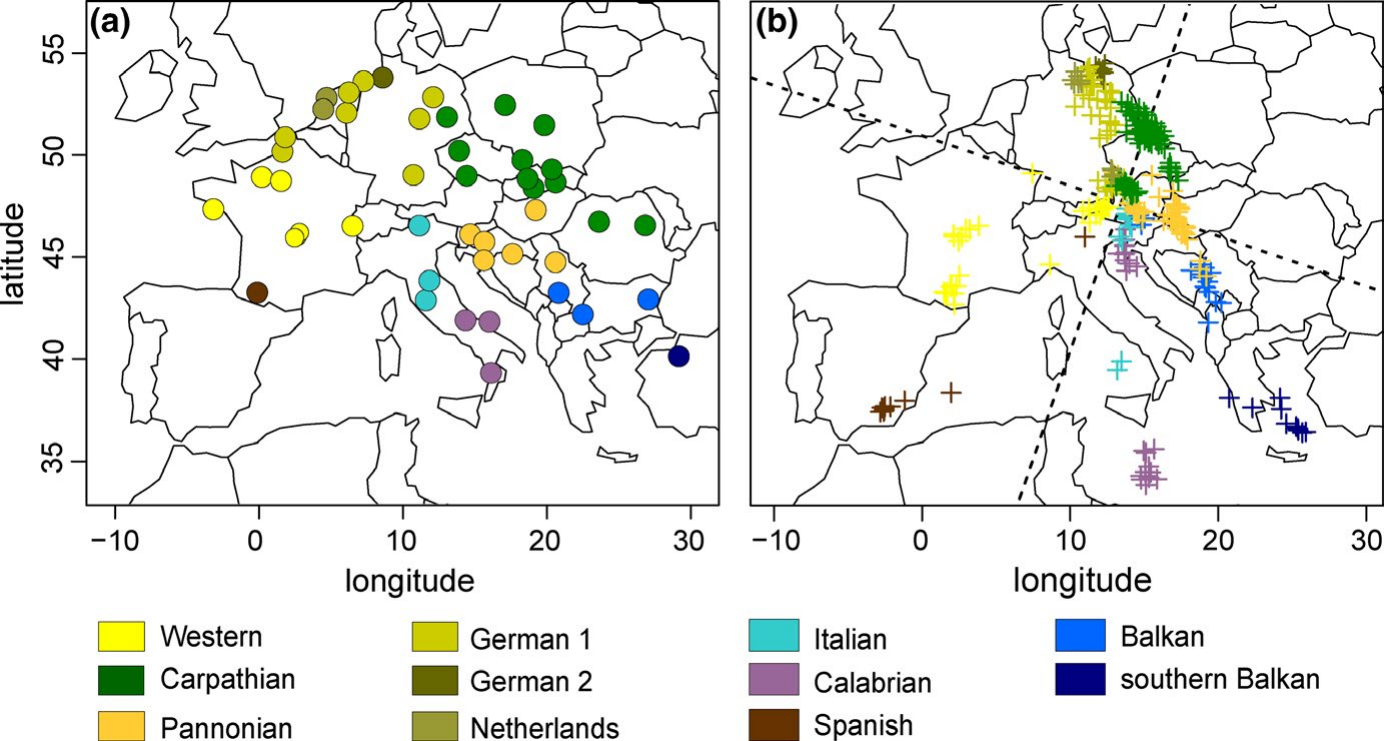
Correspondence between (a) the geographic latitude and longitude coordinates and (b) Procrustes‐transformed first two principal component (dotted lines) genetic coordinates of 316 bank voles from 48 localities in Mediterranean and central Europe, belonging to one of the 11 color‐coded populations

Marková et al. (2020a) used the population‐clustering programs BAPS v7.13 (Corander & Marttinen, 2006; Corander, Marttinen, et al., 2008; Corander, Sirén, et al., 2008) and ADMIXTURE v1.2 (Alexander et al., 2009) for determining the number of bank vole populations across Europe and for assigning the 809 individuals to each population. These assignments provided largely concordant results, inferring 10 populations across central Europe and Mediterranean, which comprised 316 individuals from 48 localities (Figure 1a).

The results of ADMIXTURE and BAPS obtained by Marková et al. (2020a) differ slightly with respect to the Apennine and Balkan peninsulas. In Italy, BAPS identified a separate population in northern Italy, while in ADMIXTURE, the Italian population exhibited almost equal amounts of admixture from surrounding populations (Spanish, Pannonian, Balkan and Calabrian) and did not form a separate cluster. In the Balkan Peninsula, ADMIXTURE separated bank voles in southeastern Europe into two distinct populations (Balkan and southern Balkan), while in BAPS, these form only one (Balkan). As a more detailed partitioning was desirable for the analyses in the present paper, we considered bank voles from northern Italy (Italian) and southern Balkans (which includes Turkey; Çolak et al., 2016; Deffontaine et al., 2005) to be separate populations, totaling up to 11 populations (Figure 1a). Therefore, in southern Europe, we recognize five Mediterranean populations: one in Spain (Spanish), two in Italy (Italian in northern Italy and Calabrian), and two in the Balkan Peninsula (Balkan and southern Balkan). As already established by Marková et al. (2020a), in the eastern part of central Europe samples were assigned to two populations (Carpathian in the north and Pannonian in the south) and in the western part to four populations: Western in the west, and German 1, German 2, and Netherland populations, located primarily in Germany and the Netherlands.

### 2.2 Population genetic diversity and structure

To evaluate geographic trends in genetic diversity, we mapped each of the 103 localities and interpolated two measures of diversity, the proportion of polymorphic loci (*P*) and the Watterson's estimator of theta (*θ*) (Marková et al., 2020a), across the species range. To perform the interpolation, we used the inverse distance weighted (IDW) method and the Geostatistical Analyst extension to ArcMap v10.6 (Redlands, CA).

To infer the evolutionary relationships among the 11 populations from central Europe and Mediterranean, we used a maximum‐likelihood method in TreeMix v1.13 (Pickrell & Pritchard, 2012) which accounts for admixture between the populations by adding migration events during the tree building process until the tree explains 99% of the variance in the data, and the admixture weights for each migration event are then estimated (Pickrell & Pritchard, 2012). To explore the correlation between population genetic variation and geography, we contrasted genetic relationships described by first two eigenvectors of principal component analysis (PCA) performed with EIGENSOFT v7.2.1 (Price et al., 2006) with the coordinates of the sample locations using a Procrustes analysis conducted in the “vegan” package in R. Procrustes analysis quantifies the correlation by rotating first two PC axes to assess similarity to the geographic distribution of the sampled locations, that is, superimposing the PCA plot of genetic variation on the geographic map (Wang et al., 2012). Significance of the associations was evaluated based on 10,000 permutations.

### 2.3 Testing historical colonization scenarios

To select among sets of possible colonization scenarios those best explaining the observed SNP variation, we used an ABC‐RF model choice approach method (Pudlo et al., 2016) analogous to that adopted by Marková et al. (2020a), but involving different populations and geographic areas. Specifically, we focused on scenarios regarding the role of Carpathian refugium and traditional Mediterranean refugia in colonization of central Europe. We used a sequential testing approach beginning with the sets of simple scenarios to infer basic relationships between the populations within particular focal areas. The winning scenarios were used in subsequent analyses of more complex population history patterns. In the first, “Balkan” dataset (Figures A2 and A3 in Appendix S1), we investigated contributions of southern Balkan (Mediterranean) and Carpathian (extra‐Mediterranean) populations in colonization of the southeastern part of Europe (Balkan and Pannonian populations) using two sets of five and seven scenarios, respectively, following increasing complexity. Because the TreeMix analyses suggested an admixed origin of the Balkan population derived from the Carpathian and southern Balkan populations (see Results), and the Pannonian population characterized by mtDNA related to the Carpathian lineage (Deffontaine et al., 2005; Filipi et al., 2015) is clustered with the Balkan population by BAPS (Marková et al., 2020a), we also included admixture scenarios. In the second, “Mediterranean” dataset (Figures A4–A6 in Appendix S1), we used three sets of five, four, and five scenarios, respectively, to investigate the relationships between the populations in areas of the traditional Mediterranean refugia, that is, Spain, Italy, and the Balkans, and including also the Pannonian population. In particular, we investigated whether the population in Italy has contributed to populations outside the Apennine peninsula or whether it is itself of admixed origin, as suggested by ADMIXTURE (Marková et al., 2020a), TreeMix and Procrustes analyses (see Results). The third, “Western” dataset (Figures A7 and A8 in Appendix S1), comprised a set of three and six scenarios, respectively, where we tested the contribution of Spanish (Mediterranean) and Carpathian (extra‐Mediterranean) refugia to the origin of the Western population with mtDNA related to Carpathian and Pannonian (Deffontaine et al., 2005; Filipi et al., 2015), but clustering with the Spanish population in the TreeMix phylogeny (see Results). For further details that relate to the tested scenarios, see “Identification of the competing historical scenarios” section in Appendix S1.

In order to identify the ancestral origin of populations and minimize the effects of recent gene flow, historical scenario tests were run using individuals from the geographic sampling localities with the least amount of admixture (Table A1 in Appendix S1). In each analysis, 10,000 datasets were simulated per scenario using DIYABC v2.1.0 (Cornuet et al., 2014). Parameter values were set up to the range of 100–200,000 diploid individuals for effective population size and 0.001–0.999 for the *r*_a_ and *r*_b_ parameters (admixture rates) with flat, noninformative uniform priors covering a broad range of demographic scenarios (Kotlík et al., 2018; Marková et al., 2020a). The parameter values for divergence times were set to the range of 10–1,500,000 for the Balkan dataset, 10–2,000,000 generations for the Mediterranean dataset and 10–600,000 generations for the Western dataset, also with flat, noninformative uniform priors. Different upper limits for divergence times were chosen to account for historical scenarios suggested by mtDNA analyses, including the early Pleistocene divergence of Calabrian lineage (Colangelo et al., 2012) and mid‐Pleistocene divergence of southern Balkan and European lineages (Çolak et al., 2016). Effective population sizes were allowed to vary among extant as well as ancestral lineages in order to model bottlenecks due to founder events during colonization. The entire set of single‐, two‐, and three‐population summary statistics available in DIYABC were applied (see Kotlík et al., 2018). Scenario selection was performed using a tree‐based machine learning method, an RF classifier implemented in the abcrf package version 1.7.1 (Pudlo et al., 2016). We generated an RF of 500 trees using the reference table of summary statistics from DIYABC simulations, adding the axes of a linear discriminant analysis (LDA) of the reference table to the original set of summary statistics (Pudlo et al., 2016). We applied the RF to predict the best scenario for our datasets and to estimate the prior error rate of the classifier and the posterior probability for the selected scenario. We ran 10 iterations of the RF analysis for each scenario choice analysis in each dataset independently to ensure consistency of the results. Finally, we simulated a 100,000 dataset under each of the three winning scenarios in DIYABC, using all available summary statistics, and applied the RF approach for parameter inference (Raynal et al., 2019). For each estimated parameter, a regression RF of 500 trees was constructed and trained on bootstrap samples of the reference table from DIYABC simulations. The predicted values of the parameter were determined by averaging over all trees (Raynal et al., 2019).

## 3 RESULTS

**FIGURE 2:**
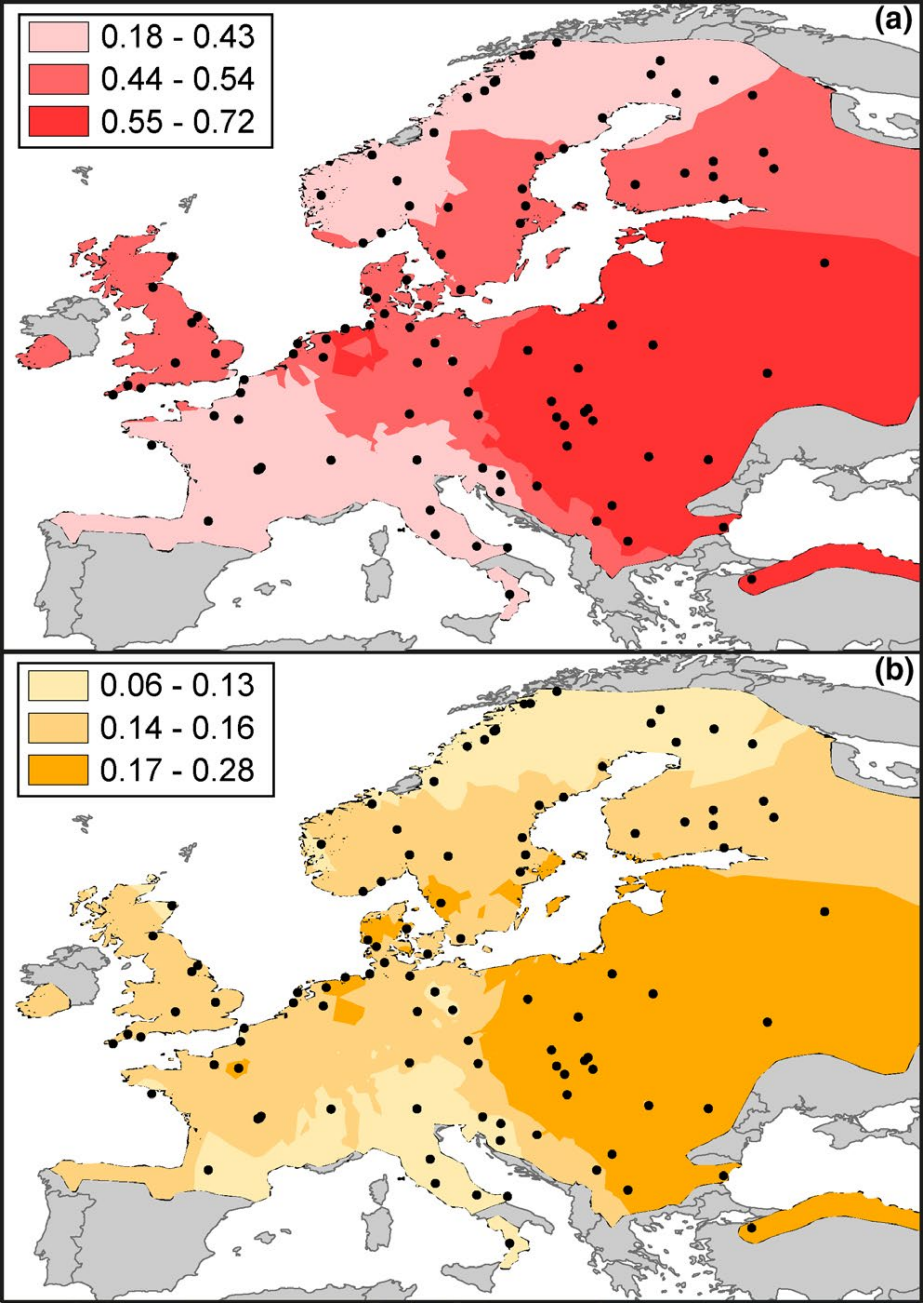
Bank vole genetic diversity across Europe represented by spatial interpolation of (a) percentage of polymorphic loci and (b) theta values (taken from Marková et al. (2020a)) for all 103 localities

The distribution of genetic diversity across the bank vole range in Europe is marked by elevated values in mid‐latitudes, particularly in the central‐eastern part of Europe, that is, around the Carpathian refugium and Eastern part of Europe (Figure 2). In contrast, the Spanish and Italian refugial locations exhibited relatively low diversity, similar to the northern apex of the range in Fennoscandia.

**FIGURE 3:**
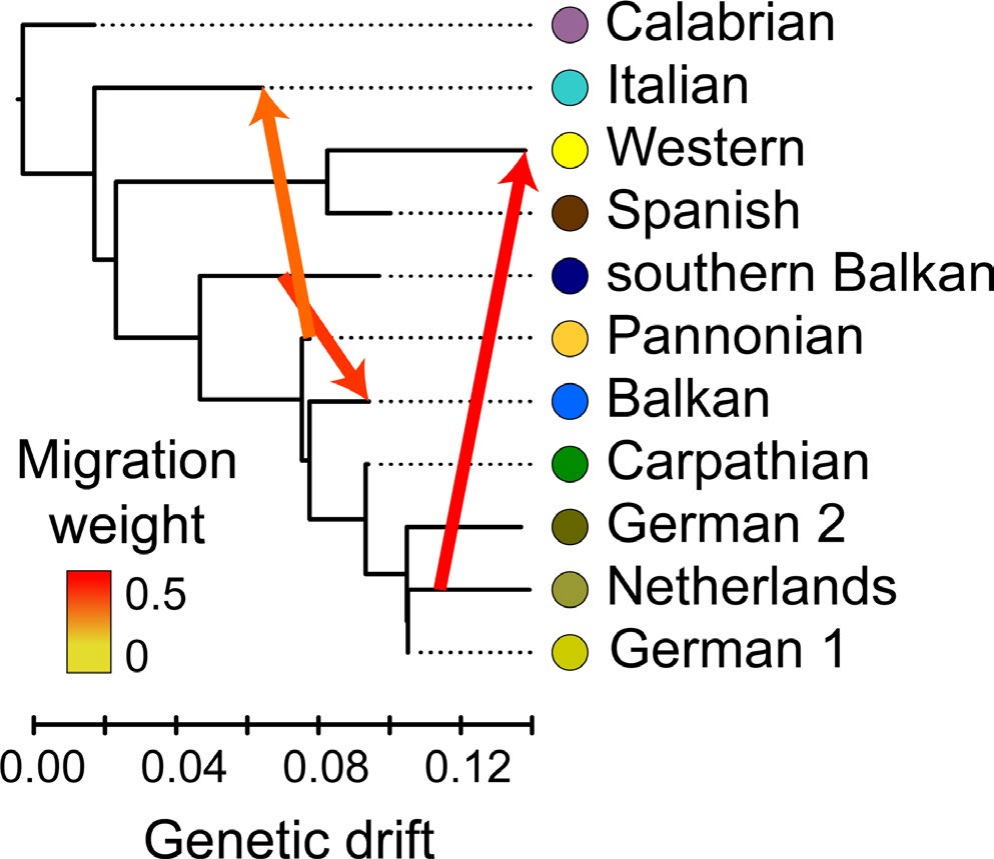
Evolutionary relationships among 11 bank vole populations from Mediterranean and central Europe (Figure 1), estimated using TreeMix with three migrations events depicted by arrows color‐coded to the inferred migration weight (admixture proportion)

According to our TreeMix analysis (Figure 3), the Mediterranean refugial populations (Calabrian, Italian, Spanish, and southern Balkan) branch off first, carrying the most divergent genomes of bank vole populations. Interestingly, the Western population did not group with the Carpathian population as it does in the mtDNA phylogeny (Figure A1 in Appendix S1), but with the Spanish population (Figure 3). Populations from northern Germany and the Netherlands (German 1, German 2, and Netherlands) group together with the Carpathian population. Adding three migration events explained 99% of the variance, with an additional migration only contributing a marginal increment. The inferred migration events (Figure 3) suggest an admixed origin of the Italian population (with 32.9% admixture from Pannonian, *SE* = 0.025, *p*‐value < 0.001), Balkan population (with 39.2% admixture from southern Balkan, *SE* = 0.026, *p*‐value < 0.001), and Western population (with 46.4% admixture from Netherlands, *SE* = 0.05, *p*‐value < 0.001).

The Procrustes analysis identified a significant similarity score between the position of the individuals in the PC space and their geographic locations (*t* = 0.79, *p*‐value < 0.001). The resulting graph shows that the populations occupy a narrower longitudinal extent and broader latitudinal extent than expected by their geographic locations, with many populations exhibiting a loose association between rotated PC axes and geographic location (Figure 1b). Individuals from the three Mediterranean nonadmixed populations (Spanish, Calabrian and southern Balkan) are further from the rest of the populations, indicating they are more genetically divergent from the rest of European populations than expected by their geographic locations (Figure 1a). Most of the remaining populations are tightly associated in central Europe and genetically closer than their geographic locations suggest (Figure 1b).

**FIGURE 4:**
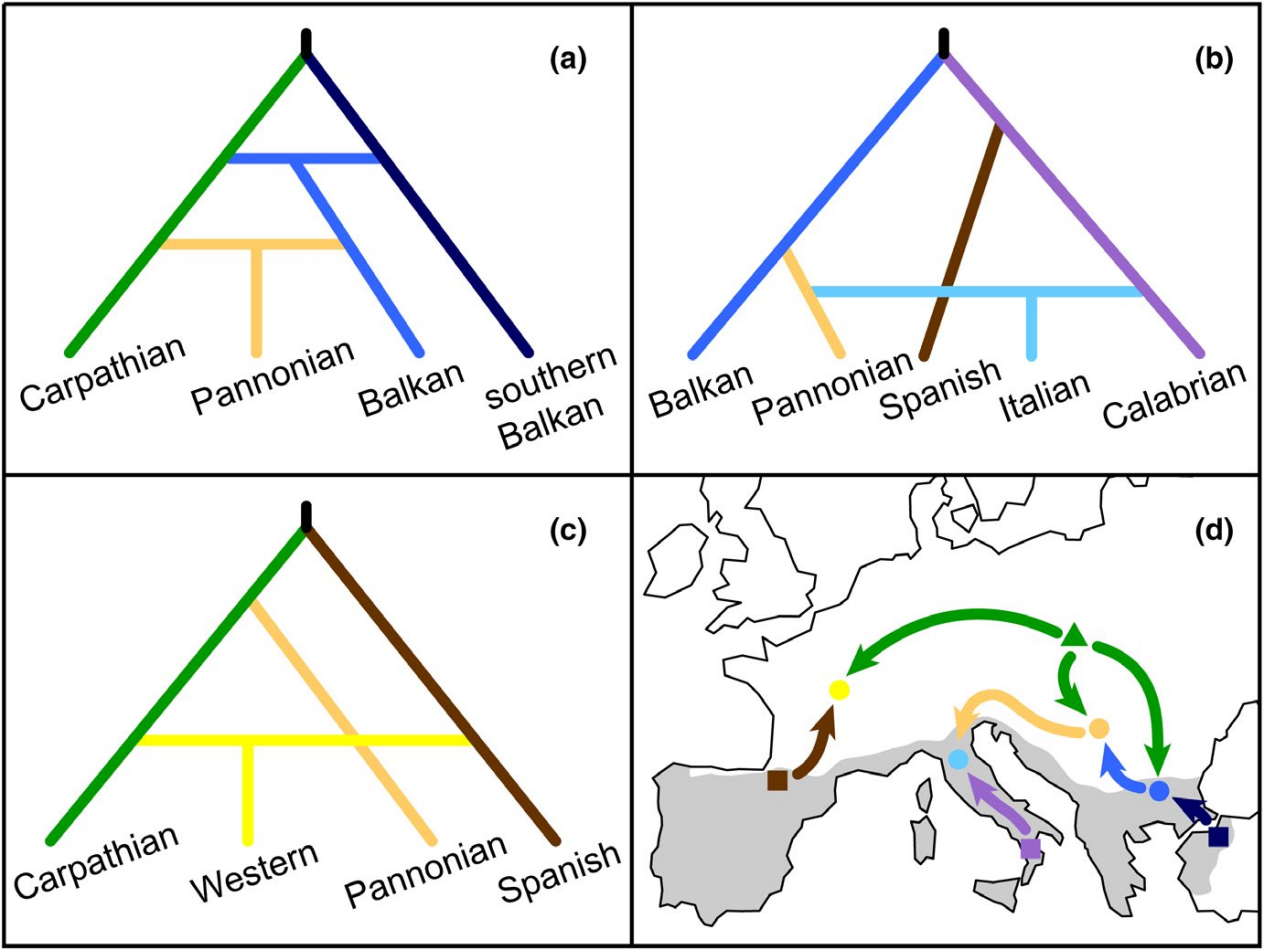
The winning scenarios from the three ABC‐RF scenario choice analyses of colonization of central Europe by bank voles from Mediterranean and Carpathian glacial refugia, each concerning a different set of populations: (a) origin of the Balkan and Pannonian populations from sequential admixture between the southern Balkan and Carpathian populations, (b) admixed origin of the Italian population from the Calabrian and Pannonian populations, and (c) admixed origin of the Western population from the Spanish and Carpathian populations. (d) A schematic map synthesizing the three scenarios. The different shapes symbolize the refugial source (triangle, Carpathian; squares, Mediterranean) and admixed populations (circles). The arrows denote the inferred genomic contributions, but do not necessarily represent the exact paths of colonization. The approximate extent of the Mediterranean region of Europe is shaded in gray

Comparisons of alternative colonization scenarios with ABC‐RF within three focal areas reveal admixed origins of the Balkan, Italian, and Western populations and suggest a double admixture origin for the Pannonian population. The best‐supported scenario in the Balkan dataset demonstrated two admixture events (Figure 4a): first, an origin of the Balkan population from admixture between the southern Balkan (46%) and Carpathian (54%) populations, and a second admixture between the Balkan (28%) and Carpathian (72%) populations to give rise to the Pannonian population (Figure 4a). In the winning scenario of the Mediterranean dataset, admixture between the Pannonian (46%) and Calabrian (54%) populations resulted in formation of the Italian population (Figure 4b). In the Western dataset, the best‐supported scenario includes an origin of the Western population by admixture between the Spanish (47%) and Carpathian (53%) populations (Figure 4c). Therefore, the migration inferred between the Carpathian/German 1/German 2/Netherlands clade and the Western population (Figure 3) is most likely a consequence of admixture among those populations, in agreement with results of the Procrustes analyses indicating their close genetic proximity (Figure 1b).

Taken together, the ABC‐RF estimates suggest bank vole populations in central Europe received around 60% of their ancestry from the Carpathian refugium, but Mediterranean refugia contributed around 40% (i.e., Spain ~25% and southern Balkans ~15%) (Table A2 in Appendix S1). There was no evidence that the Italian or Calabrian populations contributed to bank vole genomic diversity in central Europe.

Although the analyses yielded broad confidence intervals for model parameters (Tables A3–A5 in Appendix S1), the time estimates indicate that the Carpathian, southern Balkan, Calabrian, and Spanish populations diverged from the ancestral populations well before the LGM (mean divergence time of 100–200 kyr, assuming a generation time of two years; Ryabokon & Goncharova, 2006), as expected for populations representing separate LGM refugia. The estimates of 6–15 kyr for the origin of the four admixed populations (Balkan, Pannonian, Italian and Western) are consistent with the expansion from these refugia starting shortly after the LGM and extending into the early Holocene.

## 4 DISCUSSION

By evaluating previously obtained genomic data for over 800 voles in a new analysis, we provide clear evidence of both Mediterranean and extra‐Mediterranean (i.e., Carpathian) refugial contributions to the ancestry and genomic diversity of bank vole populations in central Europe. This refines conclusions based upon mtDNA alone, which suggested little Mediterranean contribution (e.g., Bilton et al., 1998; Deffontaine et al., 2005; Kotlík et al., 2006), and it is only through analysis of this extensive SNP dataset that the complex history of admixture between different refugia is revealed. Although the genomic contribution of the Carpathian refugium predominates, regional genomic diversity is strongly shaped by the patterns of repeated admixture between the four refugial populations (three Mediterranean and one Carpathian). Likely facilitated by the greater connectivity of continental populations when compared to peripheral portions of the range (e.g., Kotlík et al., 2018; Marková et al., 2020a), this history of frequent admixture in central Europe drives a pronounced mid‐latitude peak in genomic diversity.

Bank voles have been at the forefront of novel perspectives in European phylogeography, providing evidence of extra‐Mediterranean refugia (Bilton et al., 1998; Deffontaine et al., 2005; Kotlík et al., 2006), lineage replacement (Kotlík et al., 2018; Marková et al., 2020a; Searle et al., 2009), and adaptive phylogeography (Kotlík et al., 2014). The present study demonstrates the complex end‐glacial colonization history of this informative model species and underscores the importance of genomic data for hypothesis testing in phylogeography. It supports a model of genome‐wide divergence consistent with mtDNA‐based conclusions regarding major lineages (Hung et al., 2016), but reveals complex population histories and spatial patterns in the distribution of genetic diversity that would otherwise be inaccessible from a single locus estimate (Edwards et al., 2015).

### 4.1 Mediterranean genomic contributions to the end‐glacial colonization of central Europe

The presence of distinct, divergent mtDNA haplotypes in Mediterranean bank voles previously resulted in the conclusion that these populations made few genetic contributions to central European diversity (Bilton et al., 1998; Deffontaine et al., 2005; Filipi et al., 2015). Mediterranean populations appeared isolated on separate peninsulas, evolving into endemic lineages that represented most of the mitochondrial diversity of the species (Bilton et al., 1998; Deffontaine et al., 2005; Filipi et al., 2015; Kotlík et al., 2006). Our analysis of genome‐wide SNP data provides a novel perspective on the role of those Mediterranean refugia by revealing that ancestral contributions from Spanish and Balkans refugial populations represent a quarter to a half of the genomic material in admixed central European bank vole populations (Table A2 in Appendix S1). Although Carpathian ancestry still comprises the dominant genomic influence, this admixed history presents opportunities for novel allele combinations with the potential for important evolutionary consequences (see below).

Genomic data indicate the role of major mountain ranges in shaping the differential contributions from each Mediterranean refugium. For example, Spanish genomic contributions in southern France and northern and central Italy (Marková et al., 2020a) suggest that Spanish populations had to cross the Pyrenees and spread north, where they admixed with other ancestral populations (Figure 4d). However, neither the Italian nor the Calabrian populations appear to have crossed the Alps or contributed to central European diversity (Figure 4d). Instead, the Italian population received ancestry from the Pannonian populations (itself admixed between the Carpathian and Balkan populations) that entered the Italy from the northeast (e.g., along the then exposed northern Adriatic shelf), in addition to the Calabrian population from the south (Figure 4d). Thus, Mediterranean and extra‐Mediterranean populations together contributed to the complex present pattern of genetic diversity of the bank voles in Italy (Chiocchio et al., 2019). Spanish but not Italian genomic contributions to central European populations of bank voles support other findings that the Pyrenees were not as strong of a barrier to colonization of terrestrial organisms as the Alps, with the presence of Spanish haplotypes of many other forest species in northern Spain, western France, Britain, and Ireland (Petit et al., 2003). Similarly, populations from the Balkan refugium were not prevented from expanding further north by high mountains (e.g., Pyrenees and Alps) (Cooper et al., 1995; Hewitt, 1999). These landscape differences also shape genomic contributions: For example, Spanish and Balkan refugia contribute ~23% and ~14% of their genomes (Table A2 in Appendix S1), respectively, to admixed central European populations. For many central European mammals, mtDNA analyses suggest the Balkan refugia were the main source for end‐glacial colonization, including small mammals with ecology similar to bank voles (e.g., the yellow‐necked field mouse *Apodemus flavicollis*; Michaux et al., 2004). Our study suggests that high‐resolution genomic markers, broad geographic sampling, and rigorous model testing are needed to re‐examine the contribution of different refugia in other species and to evaluate the role of species traits and landscape configuration to colonization dynamics in this region.

### 4.2 Widespread genomic footprints of the Carpathian refugium

The analysis of the genomic data provides clear evidence that the Carpathian refugium has contributed the greatest share of ancestry to the admixed bank vole populations in central Europe (over 60%). Evidence from the fossil record (Horáček, 2000; Nadachowski, 1989; Nadachowski et al., 2003) and mtDNA (Deffontaine et al., 2005; Kotlík et al., 2006; Wójcik et al., 2010) suggests continuous presence of this species in the Carpathian Mountains. Detailed pollen analyses of the Carpathian Basin during the LGM suggest a widespread patchwork of suitable microhabitats: lowlands dominated by dry steppe with wet and mesic grasslands, with forest patches or scattered trees occurring in river valleys, on north‐facing hillslopes, and at moister sites of the loess plateaus (Magyari et al., 2013). Niche modeling of bank voles also supports broad climatic suitability in the region, with a greater predicted area of suitability for the Carpathian mtDNA lineage at the LGM and mid‐Holocene, compared to the other bank vole refugial lineages (Escalante et al., 2021). Survival in multiple microrefugia across the Carpathians, with relatively small populations and repeated gene flow, suggests a metapopulation scenario that would increase long‐term effective population size and reduce allele loss (Furlan et al., 2012). Supporting this hypothesis, the genomic data suggest the highest genetic diversity (*π* = 0.246; Table A1 in Appendix S1) in the Carpathian population (Figure 2) compared to all other regions. This model of geographically scattered, low‐density populations (Rull, 2009; Stewart et al., 2010; Willis & Van Andel, 2004) and the resulting patterns of genomic variation may be an appropriate fit for other species (Magri, 2008; Stojak et al., 2015) across central and northern Europe. Finally, the central location and limited dispersal barriers surrounding the Carpathian refugia appear to have made it a particularly important source of colonization for surrounding populations in all directions (Figure 4d), as well as driving the high diversity found in mid‐ and high‐latitude populations further north (Marková et al., 2020a).

### 4.3 Admixture and the geographic distribution of genomic diversity

Together, these results support a pattern of repeated admixture shaping genomic variation across the bank vole range in Europe. When the climatic conditions for temperate species in central Europe became suitable at the end of the LGM, populations began to expand and colonize newly available terrain (Hewitt, 2000). While Mediterranean populations would have had to cross the Pyrenees, the Alps and the Balkan Mountains, the Carpathian population would have had a dispersal advantage to the north (Marková et al., 2020a) but also to the south and west, where it met and admixed with the Mediterranean populations. For example, ABC‐RF analyses revealed that the Spanish population from the west admixed with Carpathian population spreading from the east and formed the Western population (Figure 4d) which is now found throughout western Europe as far as southern Britain (Kotlík et al., 2018). Similarly, populations of the southern Balkans and Carpathians admixed in the central Balkans and further admixed with the Carpathian population again in the Pannonian basin (see Figure 4d). Finally, the extended land connection between the Balkans and the Italian Peninsula during the LGM and late glacial, with climates likely suitable for the bank voles (Escalante et al., 2021; Fløjgaard et al., 2009), would have facilitated faunal and genetic exchange between the refugia (Sommer & Nadachowski, 2006) allowing the formation of the admixed Italian population (Figure 4d).

Evidence suggests admixture in central Europe has continued well past the end‐glacial colonization. ADMIXTURE results (Marková et al., 2020a) suggest mixing of populations *via* gene flow is an ongoing process in central Europe. Clearly, the German 1, German 2, and Netherland populations originated from additional, recurrent contact and admixture between adjacent populations (Western and Carpathian). In contrast, in Fennoscandia and Britain, the influx of genes from the continental populations was episodic and restricted to specific windows of connectivity by land bridge submersion (Kotlík et al., 2018; Marková et al., 2020a; Searle et al., 2009).

This history of recurrent admixture, driven by differences in connectivity across the landscape, has important implications. When compared across Europe, genomic diversity of bank vole populations was highest in mid‐latitudes around the Carpathians, Balkans, and Eastern Europe (Figure 2). ADMIXTURE results revealed a complex pattern of population structure in this region, with frequent admixture between various ancestral populations, compared to the Mediterranean populations (e.g., southern Balkan; Calabrian and Spanish) or Fennoscandia and Britain, where admixture was largely restricted to between local populations (Marková et al., 2020a). Clustering analyses (BAPS and ADMIXTURE) indicate six population clusters including subgroups of the Western and Carpathian lineages (populations in Germany and the Netherlands: German 1, German 2, and Netherlands; and Pannonian Basin: Pannonian) in central Europe, compared to only four in the Mediterranean (Marková et al., 2020a). Nested sets of historical scenario selection revealed the ancestral origin of populations and indicated at least four major historical admixture events contributed to the present populations of Europe (Figure 4). With relatively lower diversity in southern refugia, these results fit a “hotspots but not melting pots” prediction, where genetic richness at intermediate latitudes is a consequence of the admixture of divergent populations colonizing central Europe from separate refugia (Petit et al., 2003).

### 4.4 Population origin may shape differential environmental tolerances

Understanding these historical patterns of isolation and admixture is important for evaluating their impacts on evolutionary change across the bank vole range. For example, intraspecific differentiation associated with different refugial populations is linked with present‐day differences in ecological niche (Escalante et al., 2021). Bank voles emanating from separate glacial refugia may have evolved different environmental (e.g., thermal) tolerances and may be adapted to local refugial conditions. We suggest the expansion of populations with different adaptive alleles may have played a role in determining phylogeographic patterns (i.e., “adaptive phylogeography”; Kotlík et al., 2014). For example, the inferred admixture events may have involved provisioning “warm‐adapted” southern alleles with specific physiological functions to the northern populations, facilitating the Holocene expansion of the admixed populations throughout central and northern Europe (Kotlík et al., 2014). For example, the β‐globin allele β52 Cys likely dispersed postglacially with the Western bank vole lineage (Strážnická et al., 2018) and appears to confer better responses to environmentally induced oxidative stress (Kotlík et al., 2014). The admixed Western population, which has about a 47% ancestral contribution from the Spanish population, shares the β52 Cys allele with the Spanish population (Strážnická et al., 2018). We hypothesize that this allele was transferred from Spanish to Western populations and spread to western Europe and Britain where it contributed to genic and population replacement (Kotlík et al., 2014). The admixed zones located in the vicinity of the Alps, Pannonian Basin, and Balkan Mountains (Figure 4d) may also influence adaptive variation, with adaptive alleles spread throughout the admixed populations (e.g., Liepelt et al., 2002).

It has been predicted that if climate change or habitat conversion drives the loss of Mediterranean populations, we will lose large portions of the genetic diversity of the bank vole (Bilton et al., 1998). However, our results show genomic material from the Mediterranean populations (Spanish and southern Balkan) admixed with the genomes from the Carpathian refugium in different regions (southern France, Balkans, and Pannonian Basin) transferring Mediterranean alleles into the populations in central Europe. Gene flow from the Mediterranean populations may have been important during end‐glacial colonization, but genomic contributions from these populations could potentially also serve as sources of adaptive variation under future global warming. For example, such southern alleles could be present in northern populations as “pre‐adaptations,” the result of neutral gene flow in the past (Rishishwar et al., 2015). Determining the presence, origin, and significance of these alleles will clarify the role of past selective and neutral drivers of diversity. Mounting evidence suggests that the evolutionary adaptation of populations forms a key component of the response of species to changing climate, in addition to range shifts (Bradshaw & Holzapfel, 2006; Davis & Shaw, 2001; Davis et al., 2005; Parmesan, 2006). By examining genes under selection and determining their physiological functions, the bank vole system has the potential to link past population history with adaptation and helps predict the potential for future responses. This adaptive phylogeography approach may help us understand the role of adaptive differentiation in shaping population history across complex landscapes (Kotlík et al., 2014).

## 5 CONCLUSIONS

The present study emphasizes the importance of genomic data in phylogeography. Although the fourfold lower effective population size and increased mutation rates (Brown et al., 1979) of mitochondrial DNA allow it to provide key insights into phylogeographic patterns (Avise, 2000), it has long been clear that a multilocus perspective is needed to critically evaluate historical scenarios (e.g., Knowles & Maddison, 2002). Only with the application of genome‐wide SNP data, we were able to reveal the contribution of Mediterranean refugial populations to central European genomic diversity in bank voles. Our results highlight the role of repeated admixture events between genomes emanating from the Carpathian as well as southern Mediterranean refugia in shaping the high genetic diversity of bank voles in central Europe, which may have resulted in provisioning of adaptive southern alleles to northern populations. We propose that in this way, the admixture from the Mediterranean refugia may have facilitated the end‐glacial spread of the admixed populations. Detailed analyses of the admixed variation should help expand the knowledge about the impact of different climatic conditions in separated refugia on key evolutionary processes such as gene flow and selection. By linking this variation to genes and phenotypes (Boutet et al., 2009; Kotlík et al., 2014; Strážnická et al., 2018), we can open new avenues for future research of the evolutionary adaptation to physiological challenges during climate change (Clarke, 1993; Kotlík et al., 2008; Parmesan, 2006).

## CONFLICT OF INTEREST

The authors declare no conflict of interest.

## AUTHOR CONTRIBUTIONS

**Michaela Horníková:** Investigation (lead); Writing‐original draft (equal); Writing‐review & editing (equal). **Silvia Marková:** Investigation (supporting); Writing‐original draft (equal); Writing‐review & editing (equal). **Hayley C Lanier:** Investigation (supporting); Writing‐original draft (equal); Writing‐review & editing (equal). **Jeremy B. Searle:** Conceptualization (supporting); Writing‐review & editing (equal). **Petr Kotlik:** Conceptualization (lead); Investigation (supporting); Writing‐original draft (equal); Writing‐review & editing (equal).

## Supporting information

Appendix S1

## Data Availability

This study uses publicly available data from Dryad (Marková et al., 2020b), available at: https://doi.org/10.5061/dryad.sbcc2fr34.
