# Supplementary material for: A dynamic history of admixture from Mediterranean and Carpathian glacial refugia drives genomic diversity in the bank vole
Source: Ecol Evol. 2021 May 25;11(12):8215–25. doi: 10.1002/ece3.7652 (PMC8216894; doi:10.1002/ece3.7652)
Supplement: Supplementary file 1 — Appendix S1 [file ECE3-11-8215-s001.zip › ece37652-sup-0009-Appendix.docx]

**APPENDIX**

**METHODS**

**Identification of the competing historical scenarios**

In order to decrease the computational complexity of the ABC-RF analyses, we divided the populations into datasets within central and southern Europe: (1) the Balkan Peninsula, (2) the Mediterranean and (3) western Europe. As we are interested in the role of Mediterranean and Carpathian refugia in postglacial recolonization, each of the three datasets comprised populations that could originate to varying degree from these refugia.

**Balkan dataset.** In the first dataset, we focused on the Balkan Peninsula and adjacent areas to resolve the relationship between the Balkan, Carpathian, and Pannonian populations. In the mtDNA analyses, the lineages from the southern Balkans and the main Balkans are united into a clade with early divergence from other European lineages (Deffontaine et al., 2005; Çolak et al., 2016). The Balkan lineages also group with the Italian and Spanish lineages, supporting little Mediterranean contribution to the recolonization of central and northern Europe (Bilton et al., 1998). However, the spatially explicit BAPS analysis of nuclear SNP data recognized the Pannonian and Balkan populations as one unit (Marková et al., 2020a) and many localities from the Pannonian population show considerable admixture from the Balkan population (Marková et al., 2020a). Furthermore, the TreeMix analysis suggested admixed origin of the Balkan population derived from the Carpathian and southern Balkan populations (Figure 3). The ADMIXTURE analysis also revealed a subtle trace of Carpathian ancestry in the Balkan population (Marková et al., 2020a), suggesting the Carpathian population spread southwards towards the Balkan Peninsula. Therefore, we compared competing scenarios for the Balkan Peninsula to understand the role of the Carpathian refugium in recolonization of south-eastern Europe.

To establish the basic relationships in the Balkans we first compared a set of simple no-admixture scenarios (Figure A2). The winning scenario 5, which supports common Balkan and Pannonian ancestry descended from the Carpathian population (Figure A2), was then included as scenario 1 into the second set of analyses examining the extent to which the Balkan and Pannonian populations were admixed in its origin (Figure A3).

**Mediterranean dataset.** In the second dataset, analyses were intended to establish the origin of the populations in Italy and to uncover if the Italian population contributed to the colonization of areas outside the Apennine peninsula. Previous mtDNA analyses revealed three divergent lineages in Italy (Filipi et al., 2015; Figure A1). First, the Calabrian lineage diverged in the early Pleistocene, and since then remained isolated in southern Italy (Colangelo et al., 2012). Then an additional two lineages in central Italy diverged in the late Pleistocene (Colangelo et al., 2012), one of them being closely related to the Balkan and Spanish lineages (Figure A1). Similarly, the analyses based on nuclear SNP data recognize the Calabrian population as the most divergent (Figure 3), but with inconsistent results for the rest of Italy. While the spatially explicit BAPS analysis groups bank voles from central and northern Italy into a distinct population (Marková et al., 2020a), the TreeMix (Figure 3), Procrustes (Figure 1) and Admixture analyses suggest an admixed origin of the bank voles in those areas, with considerable contribution from the Spanish, Balkan, Pannonian and also the Calabrian populations (Marková et al., 2020a). To understand this history, we compared competing scenarios evaluating the relationships between the Italian and other Mediterranean populations.

In the first analyses, we compared five simple no-admixture scenarios (Figure A4). The winning scenario 4, supporting an early divergence of the Spanish population then the Italian population from the Calabrian population, was subsequently used in the second set of analyses testing if admixture events played a role in forming the Italian population (Figure A5). Then we used the winning scenario 2, assuming earlier divergence of the Balkan population and an origin of the Italian population by admixture between the Spanish and Calabrian populations (Figure A5), in the third round of testing. Here we analysed five scenarios testing if the Pannonian population was involved in the colonization history of central Italy, but considering also the possibility that populations in Italy have contributed to populations outside the Apennine peninsula (scenarios 4 and 5; Figure A6).



**Western dataset.** The third dataset evaluates the colonization history of western Europe. Here, we focused on the relationships between the Pannonian and Western populations and the importance of the Spanish and Carpathian populations for recolonization of the area. In the mtDNA analyses, the Pannonian and Western lineages are grouped in one clade, closely related to the Carpathian lineage (Figure A1; Filipi et al., 2015; Marková et al., 2020a). Moreover, in the TreeMix analysis of nuclear SNP data, the Western population shows considerable admixture from the Netherlands population that itself is related with the German 1, German 2 and Carpathian populations (Figure 3). However, the TreeMix analysis also indicates that the Western population shares ancestry with the Spanish population (Figure 3), necessitating the inclusion of the Spanish population in the analysis.

We first compared three no-admixture scenarios (Figure A7). The winning scenario 3, supporting a common Western and Spanish ancestry, was subsequently used as scenario 1 in the second set of analyses that included six scenarios comparing a simple demographic history with those involving possible admixture events (Figure A8).

**TABLE A1** The origin of population samples used in the ABC-RF analyses, with genetic diversity measures for each population (as estimated in Marková et al., 2020a).

| Population | Locality | Country | Lat. | Long. | *N* | *P* | *π (SD)* | *θ (SD)* |
| --- | --- | --- | --- | --- | --- | --- | --- | --- |
| Balkan | Ponikve | N. Macedonia | 42.17 | 22.50 | 8 | 0.583 | 0.103 (0.052) | 0.176 (0.062) |
| southern Balkan | Bursa | Turkey | 40.12 | 29.17 | 10 | 0.527 | 0.135 (0.067) | 0.148 (0.050) |
| Carpathian | Frýdek-Místek | Czech Rep. | 49.70 | 18.38 | 10 | 0.745 | 0.246 (0.122) | 0.210 (0.070) |
| Spanish | Armendarits | France | 43.25 | -0.12 | 10 | 0.321 | 0.062 (0.031) | 0.091 (0.031) |
| Western | Vimoutiers | France | 48.92 | 0.20 | 9 | 0.713 | 0.218 (0.109) | 0.207 (0.071) |
| Pannonian | Ócsa | Hungary | 47.30 | 19.22 | 7 | 0.625 | 0.201 (0.102) | 0.196 (0.072) |
| Calabrian | Calabria | Italy | 39.30 | 16.12 | 10 | 0.362 | 0.093 (0.046) | 0.102 (0.034) |
| Italian | Trentino | Italy | 46.54 | 11.13 | 10 | 0.199 | 0.007 (0.004) | 0.056 (0.019) |

*N*, number of individuals; *P*, proportion of polymorphic loci; *π*, nucleotide diversity; *θ*, Watterson’s estimator of theta per site.

**TABLE A2** Mediterranean (southern Balkan, Spanish and Calabrian) *versus* extra-Mediterranean (Carpathian) ancestral contributions to the bank vole populations based on the results of ABC-RF analyses. Where a population of admixed ancestry contributed to the origin of a new population (Figure 4; Tables A3–A5), the percentages reflect the cumulative ancestral contributions to the new population.

| Population | Geographical region | Admixture | Ancestral contribution (%) | | | |
| --- | --- | --- | --- | --- | --- | --- |
|  |  |  | Carpathian | S Balkan | Spanish | Calabrian |
| Balkan | Mediterranean | Carpathian/southern Balkan | 54.0 | 46.0 | 0.0 | 0.0 |
| Pannonian | central Europe | Carpathian/Balkan | 71.9 | 28.1 | 0.0 | 0.0 |
| Western | central Europe | Carpathian/Spanish | 53.0 | 0.0 | 47.0 | 0.0 |
| Italian | Mediterranean | Pannonian/Calabrian | 33.1 | 12.9 | 0.0 | 54.0 |
| Contribution to admixed populations in central Europe (%) | | | 62.5 | 14.0 | 23.5 | 0.0 |

**TABLE A3** Random forest parameter estimates from the Balkan datasets (Figure A3, scenario 7), with the associated 95% Bayesian confidence intervals defined by the 0.025 and 0.975 quantiles (*Q*) of the posterior distribution. Units are number of individuals for effective population size (*N*) and number of generations for divergence time (*t*) parameters.

| Parameter | Mean | *Q*_0.025_ | *Q*_0.975_ |
| --- | --- | --- | --- |
| *N*_Carpathian_ | 40,928 | 3,429 | 175,751 |
| *N*_southern Balkan_ | 144,910 | 64,678 | 198,350 |
| *N*_Balkan_ | 97,383 | 13,562 | 189,591 |
| *N*_Pannonian_ | 118,456 | 36,718 | 197,798 |
| *N*_ancestral_ | 123,822 | 4,029 | 197,508 |
| *t*_1_ _(origin of Pannonian_ *_via_* _admixture between Carpathian and Balkan)_ | 18,521 | 297 | 54,074 |
| *t*_2_ _(origin of Balkan_ *_via_* _admixture between southern Balkan and Carpathian)_ | 22,694 | 7,603 | 74,685 |
| *t*_3_ _(divergence from ancestor)_ | 203,366 | 86,789 | 507,493 |
| *r*_a_ _(admixture rate to Pannonian from Balkan)_ | 0.61 | 0.10 | 0.96 |
| *r*_b_ _(admixture rate to Balkan from Carpathian)_ | 0.54 | 0.11 | 0.94 |

**TABLE A4** Random forest parameter estimates for the Mediterranean datasets (Figure A6, scenario 2), details are same as in the caption of Table A3.

| Parameter | Mean | *Q*_0.025_ | *Q*_0.975_ |
| --- | --- | --- | --- |
| *N*_Balkan_ | 67,267 | 8,104 | 185,768 |
| *N*_Spanish_ | 131,721 | 62,462 | 193,307 |
| *N*_Calabrian_ | 100,650 | 22,289 | 191,120 |
| *N*_Italian_ | 100,584 | 13,953 | 195,516 |
| *N*_Pannonian_ | 81,908 | 18,390 | 195,487 |
| *N*_ancestral_ | 115,034 | 6,050 | 199,522 |
| *t*_1_ _(origin of Italian_ *_via_* _admixture between Calabrian and Pannonian)_ | 31,243 | 1,223 | 112,076 |
| *t*_2_ _(Pannonian divergence from Balkan)_ | 136,915 | 10,849 | 388,840 |
| *t*_3_ _(Spanish divergence from Calabrian)_ | 438,190 | 164,657 | 820,005 |
| *t*_4_ _(divergence from ancestor)_ | 404,340 | 160,808 | 840,355 |
| *r*_a_ _(admixture rate to Italian from Calabrian)_ | 0.54 | 0.10 | 0.94 |

**TABLE A5** Random forest parameter estimates for the Western datasets (Figure A8, scenario 4), details are same as in the caption of Table A3.

| Parameter | Mean | *Q*_0.025_ | *Q*_0.975_ |
| --- | --- | --- | --- |
| *N*_Western_ | 71,537 | 12,741 | 179,811 |
| *N*_Spanish_ | 140,029 | 22,173 | 197,941 |
| *N*_Carpathian_ | 42,467 | 3,031 | 172,191 |
| *N*_Pannonian_ | 62,507 | 14,903 | 157,626 |
| *N*_ancestral_ | 123,785 | 15,509 | 196,557 |
| *t*_1_ _(origin of Western_ *_via_* _admixture between Carpathian and Spanish)_ | 13,247 | 700 | 46,028 |
| *t*_2_ _(Pannonian divergence from Carpathian)_ | 41,453 | 8,383 | 111,566 |
| *t*_3_ _(divergence from ancestor)_ | 205,508 | 61,596 | 492,042 |
| *r*_a_ _(admixture rate to Western from Spanish)_ | 0.47 | 0.22 | 0.84 |

**FIGURE LEGENDS APPENDIX**

**FIGURE A1** Schematic phylogeography of bank vole mtDNA lineages and the assignment of central European and Mediterranean localities to those lineages as previously described (Deffontaine et al., 2005; Filipi et al., 2015; Marková et al., 2020a). The Eastern lineage, distributed in Fennoscandia, eastern Europe and Siberia, has made little contribution to central Europe (Marková et al., 2020a) and therefore is not a focus of the present study.

**FIGURE A2** Comparison of the first set of scenarios testing the colonization of the Balkan Peninsula using ABC-RF. (a) Alternative no-admixture scenarios for the colonization of south-eastern Europe from Mediterranean and Carpathian refugia. Scenario 5 (highlighted) was chosen as the most likely scenario in all 10 random forest iterations, with the mean posterior probability estimated as 0.69 (*SD* = 0.01). The mean prior error rate of the RF classifier was 0.05 (*SD* = 0.00). (b) Projection of the datasets simulated under each scenario and of the observed dataset (black asterisk) on the first two linear discriminant analysis (LDA) axes. Note that depictions of different colour may overlap.

**FIGURE A3** Comparison of the second set of scenarios testing colonization of the Balkan Peninsula by ABC-RF. (a) The winning scenario from the previous analyses (Figure A2) was compared to six alternative scenarios modelling an admixed origin of the Balkan population (scenarios 2–4, admixture rate *r*_a_), the Pannonian population (scenario 5, admixture rate *r*_a_), or both (scenario 6, admixture rate *r*_a_). Scenario 7 assumes a double admixture event, where the Balkan population originates by admixture of the Carpathian and southern Balkan populations (admixture rate *r*_b_) and the Pannonian population originates by admixture of the admixed Balkan and Carpathian populations (admixture rate *r*_a_). Scenario 7 (highlighted) was chosen as the most likely in eight out of 10 random forest iterations (scenario 6 was selected twice), with the mean posterior probability estimated as 0.54 (*SD* = 0.03). The mean prior error rate of the RF classifier was 0.22 (*SD* = 0.00). (b) Projection of the datasets simulated under each scenario and of the observed dataset (black asterisk) on the first two linear discriminant analysis (LDA) axes. Note that depictions of different colour may overlap.

**FIGURE A4** Comparison of the first set of scenarios testing the relationship of Mediterranean populations with ABC-RF. (a) Alternative no-admixture scenarios among four populations of Mediterranean origin. Scenario 4 (highlighted) was chosen as the most likely scenario in all 10 random forest iterations, with the mean posterior probability estimated as 0.63 (*SD* = 0.03). The mean prior error rate of the RF classifier was 0.05 (*SD* = 0.00). (b) Projection of the datasets simulated under each scenario and of the observed dataset (black asterisk) on the first two linear discriminant analysis (LDA) axes. Note that depictions of different colour may overlap.

**FIGURE A5** Comparison of the second set of scenarios testing the relationship of Mediterranean populations with ABC-RF. (a) The winning no-admixture scenario from previous analyses (Figure A4) was compared to three scenarios modelling the admixed origin of the Italian population (scenarios 2–4, admixture rate *r*_a_). Scenario 2 (highlighted) was chosen as the most likely scenario in all 10 random forest iterations, with the mean posterior probability estimated as 0.70 (*SD* = 0.05). The mean prior error rate of the RF classifier was 0.07 (*SD* = 0.00). (b) Projection of the datasets simulated under each scenario and of the observed dataset (black asterisk) on the first two linear discriminant analysis (LDA) axes. Note that depictions of different colour may overlap.

**FIGURE A6** Comparison of the third set of scenarios testing the relationship of Mediterranean populations with ABC-RF. The Pannonian population was included to test its involvement in the colonization history of central Italy. (a) The winning scenario from the previous analyses (Figure A5) was modified by adding the Pannonian population and compared to two scenarios modelling an admixed origin of the Italian population: admixture between the Pannonian and Calabrian populations (scenario 2, admixture rate *r*_a_), the Pannonian and Spanish populations (scenario 3, admixture rate *r*_a_) and two scenarios assuming a double admixture event (scenarios 4 and 5). Both scenarios assume the origin of the Italian population by admixture between the Spanish and Calabrian populations (admixture rate *r*_b_) as in the first scenario, scenario 4 then assumes an admixed origin of the Pannonian population by admixture between the Italian and Balkan populations (admixture rate *r*_a_) and scenario 5 assumes an origin of the Balkan population by admixture between the Italian and Pannonian populations (admixture rate *r*_a_). Scenario 2 (highlighted) was chosen as the most likely scenario in all 10 random forest iterations, with the mean posterior probability estimated as 0.91 (*SD* = 0.01). The mean prior error rate of the RF classifier was 0.05 (*SD* = 0.00). (b) Projection of the datasets simulated under each scenario and of the observed dataset (black asterisk) on the first two linear discriminant analysis (LDA) axes. Note that depictions of different colour may overlap.

FIGURE A7 Comparison of the first set of scenarios testing the colonization of western Europe using ABC-RF. (a) Alternative no-admixture scenarios testing the relationship of the four populations (Western, Spanish, Pannonian and Carpathian) assumed to colonize western Europe from Mediterranean and Carpathian refugia. Scenario 3 (highlighted) was chosen as the most likely scenario in all 10 random forest iterations, with the mean posterior probability estimated as 0.73 (*SD* = 0.02). The mean prior error rate of the RF classifier was 0.03 (*SD* = 0.00). (b) Projection of the datasets simulated under each scenario and of the observed dataset (black asterisk) on the first two linear discriminant analysis (LDA) axes. Note that depictions of different colour may overlap.

FIGURE A8 Comparison of the second set of scenarios testing the colonization of western Europe using ABC-RF. (a) The winning scenario from the previous analyses (Figure A7) is compared to the three scenarios modelling an admixed origin of the Western population (scenarios 2–4, admixture rate *r*_a_) and two scenarios assuming double admixture events (scenarios 5 and 6). Scenario 5 assumes the initial origin of the Western population by admixture between the Carpathian and Spanish populations (admixture rate *r*_b_) and then an origin of the Pannonian population by admixture between the Western and Carpathian populations (admixture rate *r*_a_), while scenario 6 assumes the initial admixture event between the Carpathian and Spanish populations lead to the origin of the Pannonian population (admixture rate *r*_b_), and the second admixture event between the Pannonian and Carpathian populations (admixture rate *r*_a_) leads to the origin of the Western population. Scenario 4 (highlighted) was chosen as the most likely scenario in all 10 random forest iterations, with the mean posterior probability estimated as 0.60 (*SD* = 0.02). The mean prior error rate of the RF classifier was 0.18 (*SD* = 0.00). (b) Projection of the datasets simulated under each scenario and of the observed dataset (black asterisk) on the first two linear discriminant analysis (LDA) axes. Note that depictions of different colour may overlap.
