# Supplementary figures and images for: A dynamic history of admixture from Mediterranean and Carpathian glacial refugia drives genomic diversity in the bank vole
Source: Ecol Evol. 2021 May 25;11(12):8215–25. doi: 10.1002/ece3.7652 (PMC8216894; doi:10.1002/ece3.7652)

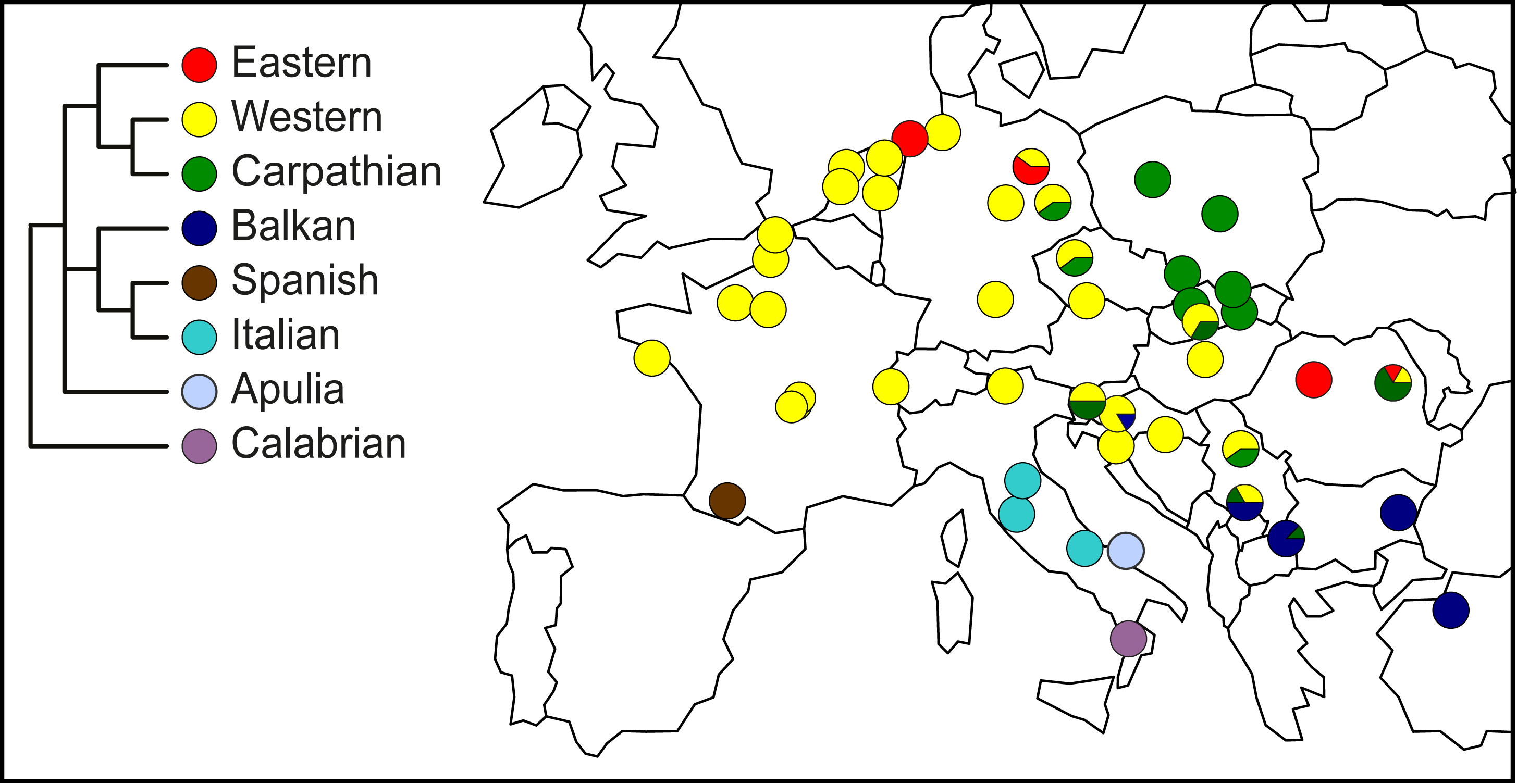

Supplement: Supplementary file 1 — Appendix S1 [file ECE3-11-8215-s001.zip › ece37652-sup-0001-FigA1.tif]

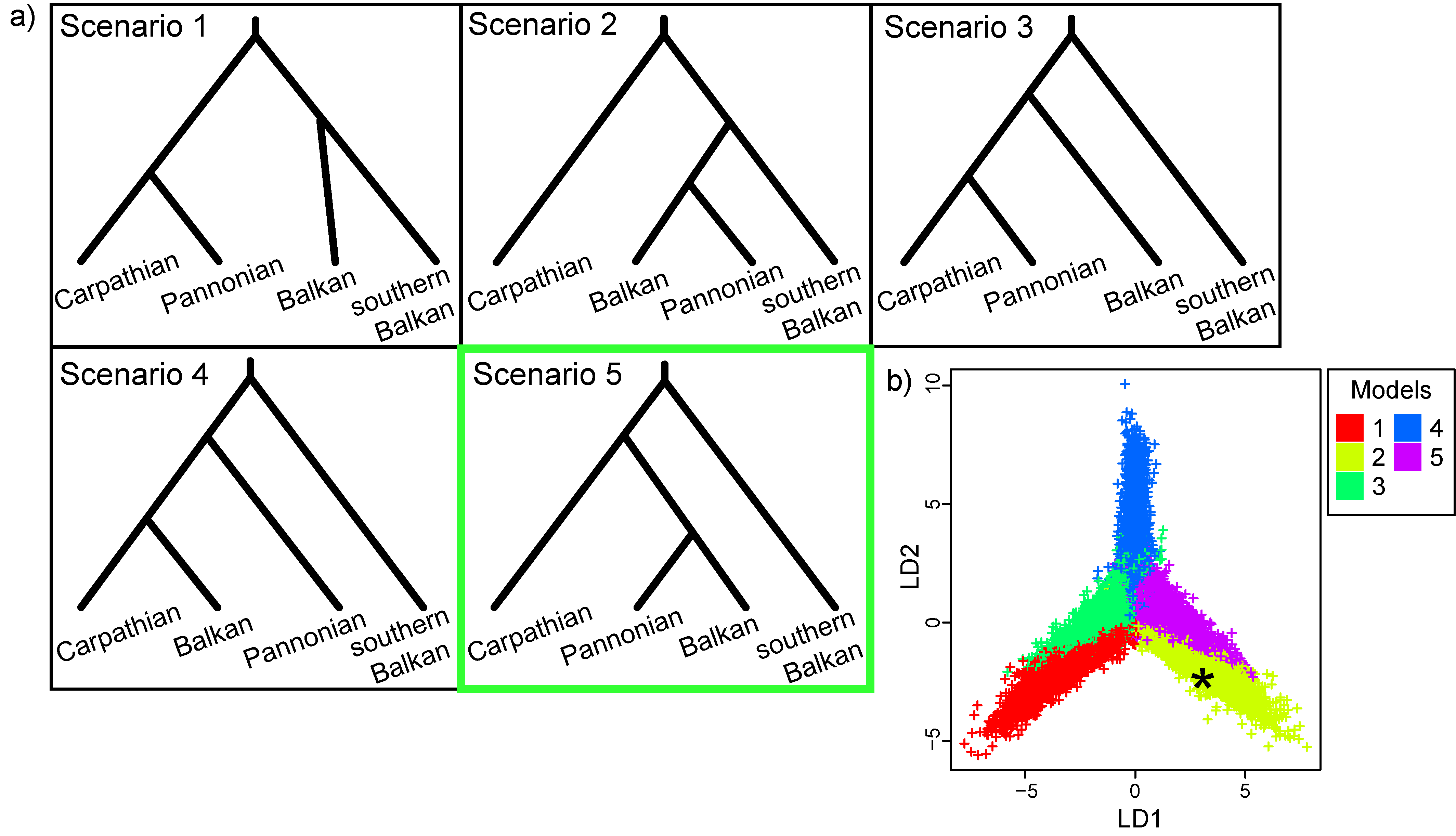

Supplement: Supplementary file 1 — Appendix S1 [file ECE3-11-8215-s001.zip › ece37652-sup-0002-FigA2.tif]

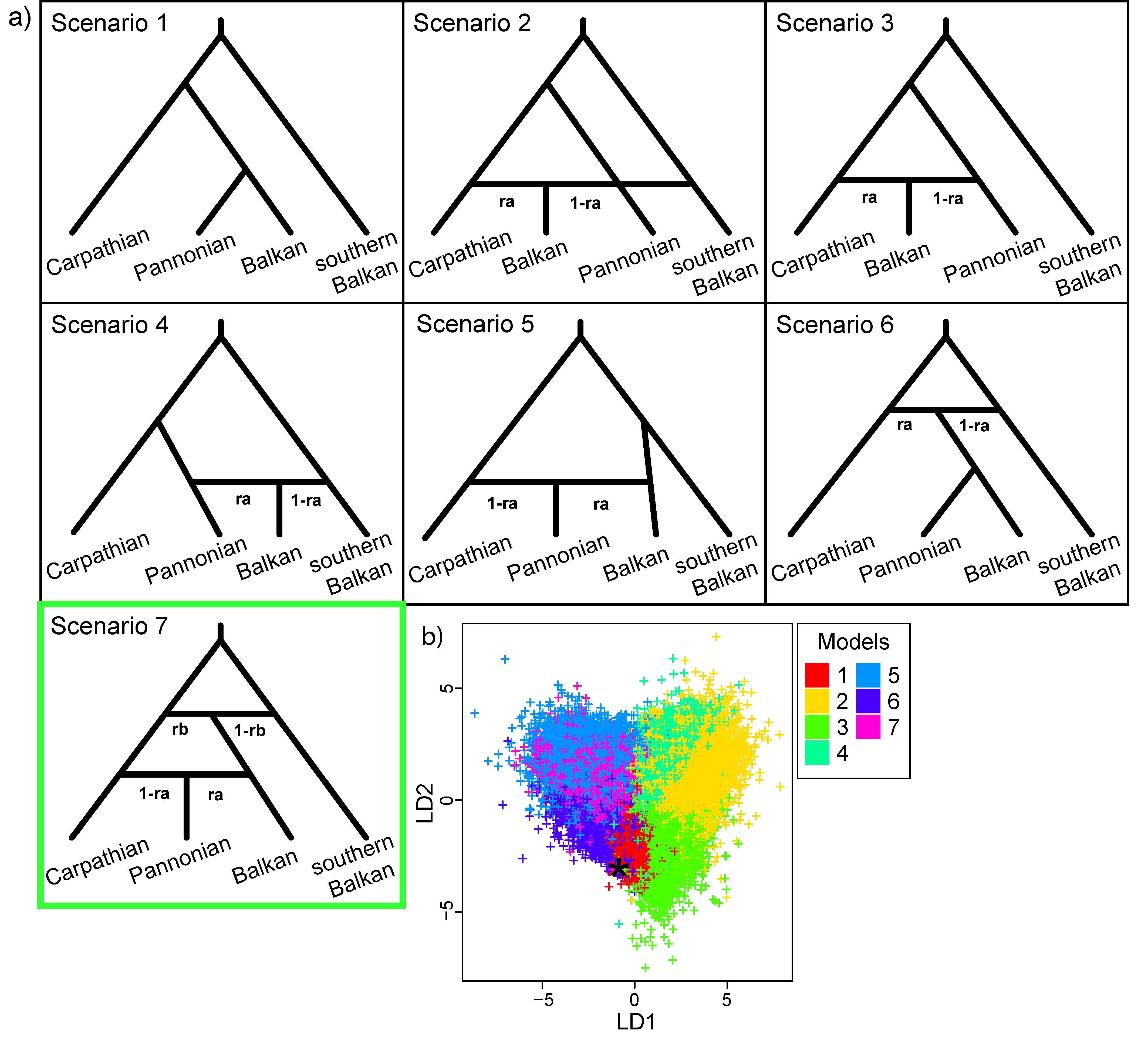

Supplement: Supplementary file 1 — Appendix S1 [file ECE3-11-8215-s001.zip › ece37652-sup-0003-FigA3.tif]

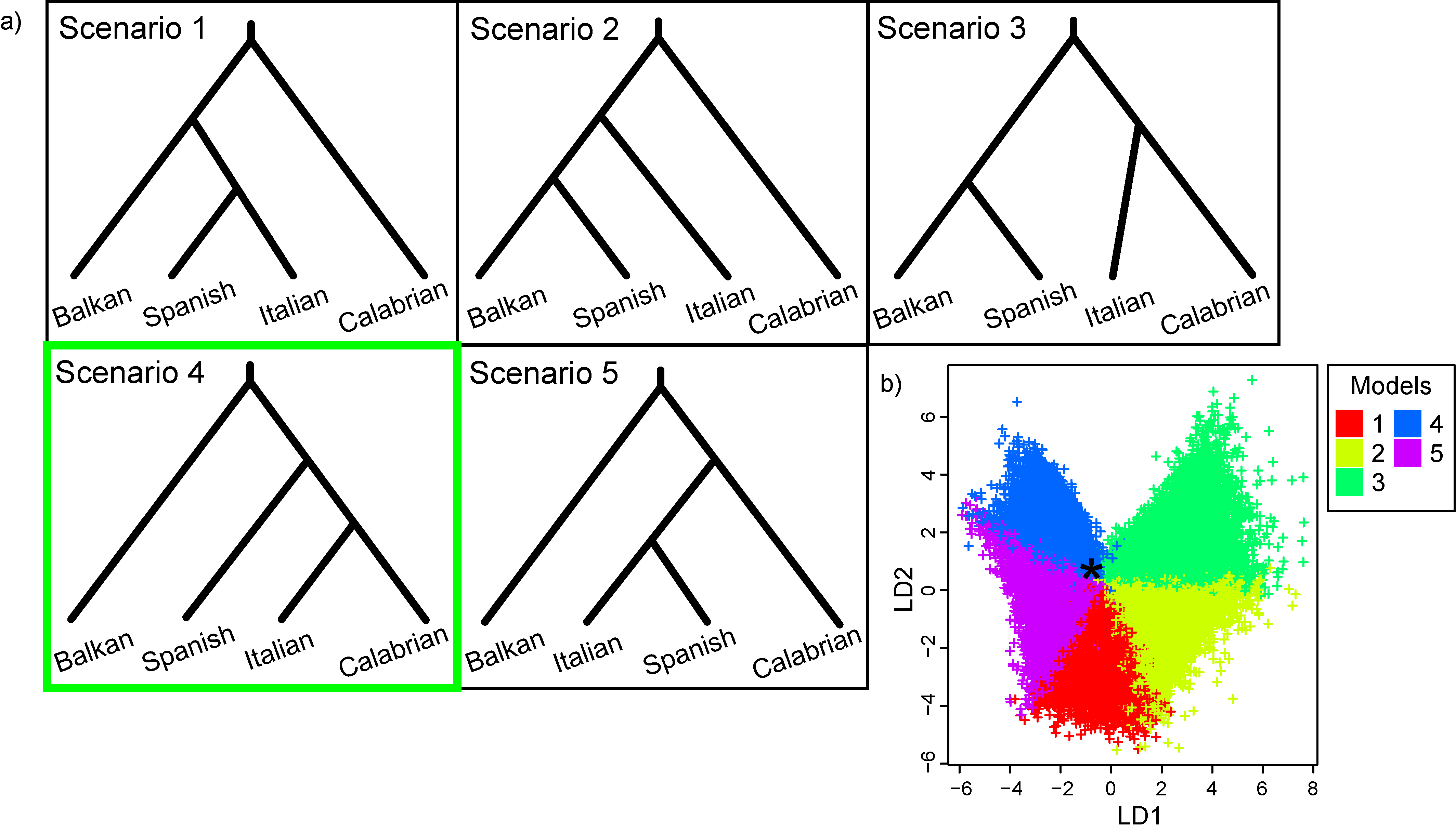

Supplement: Supplementary file 1 — Appendix S1 [file ECE3-11-8215-s001.zip › ece37652-sup-0004-FigA4.tif]

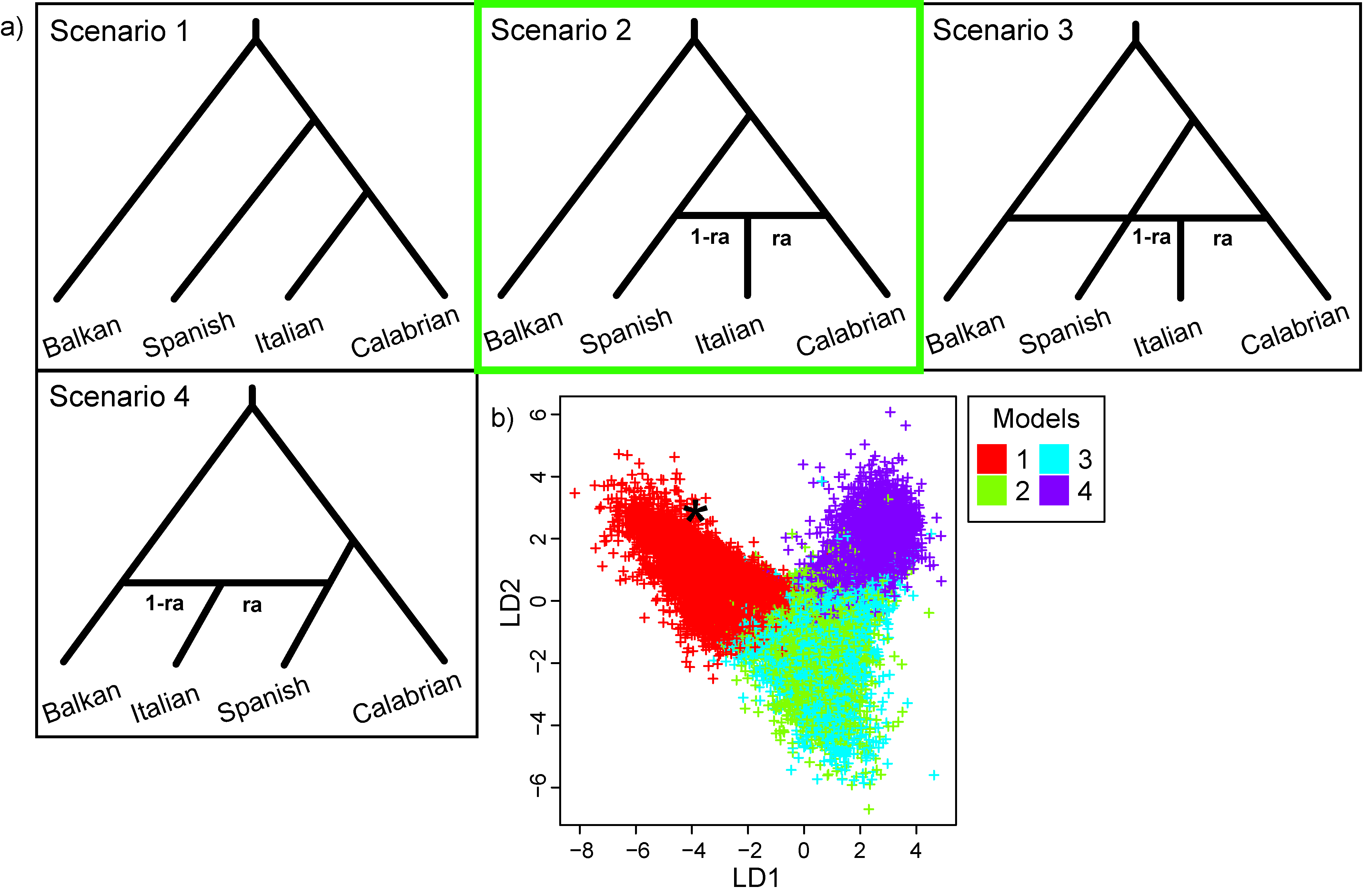

Supplement: Supplementary file 1 — Appendix S1 [file ECE3-11-8215-s001.zip › ece37652-sup-0005-FigA5.tif]

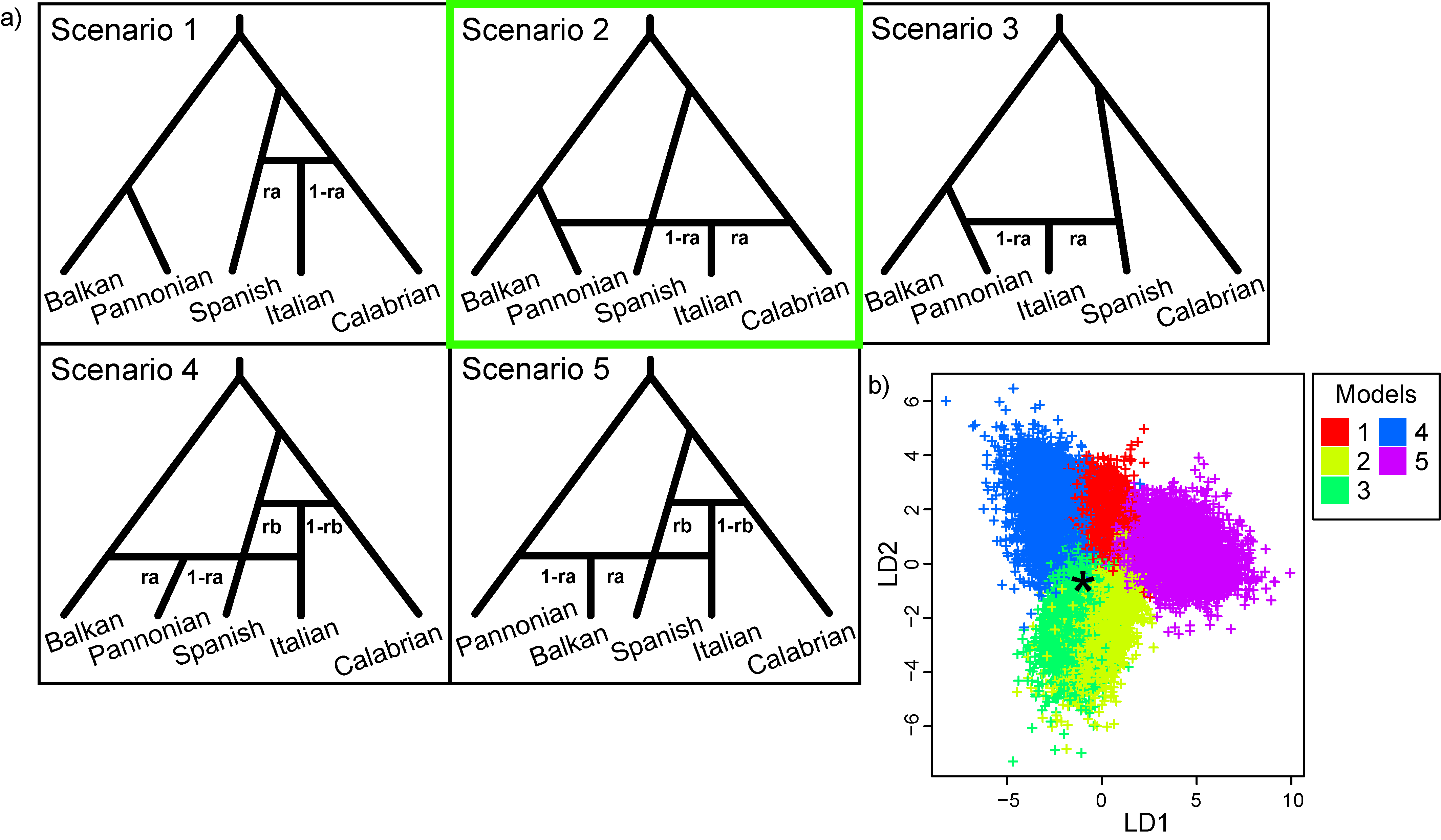

Supplement: Supplementary file 1 — Appendix S1 [file ECE3-11-8215-s001.zip › ece37652-sup-0006-FigA6.tif]

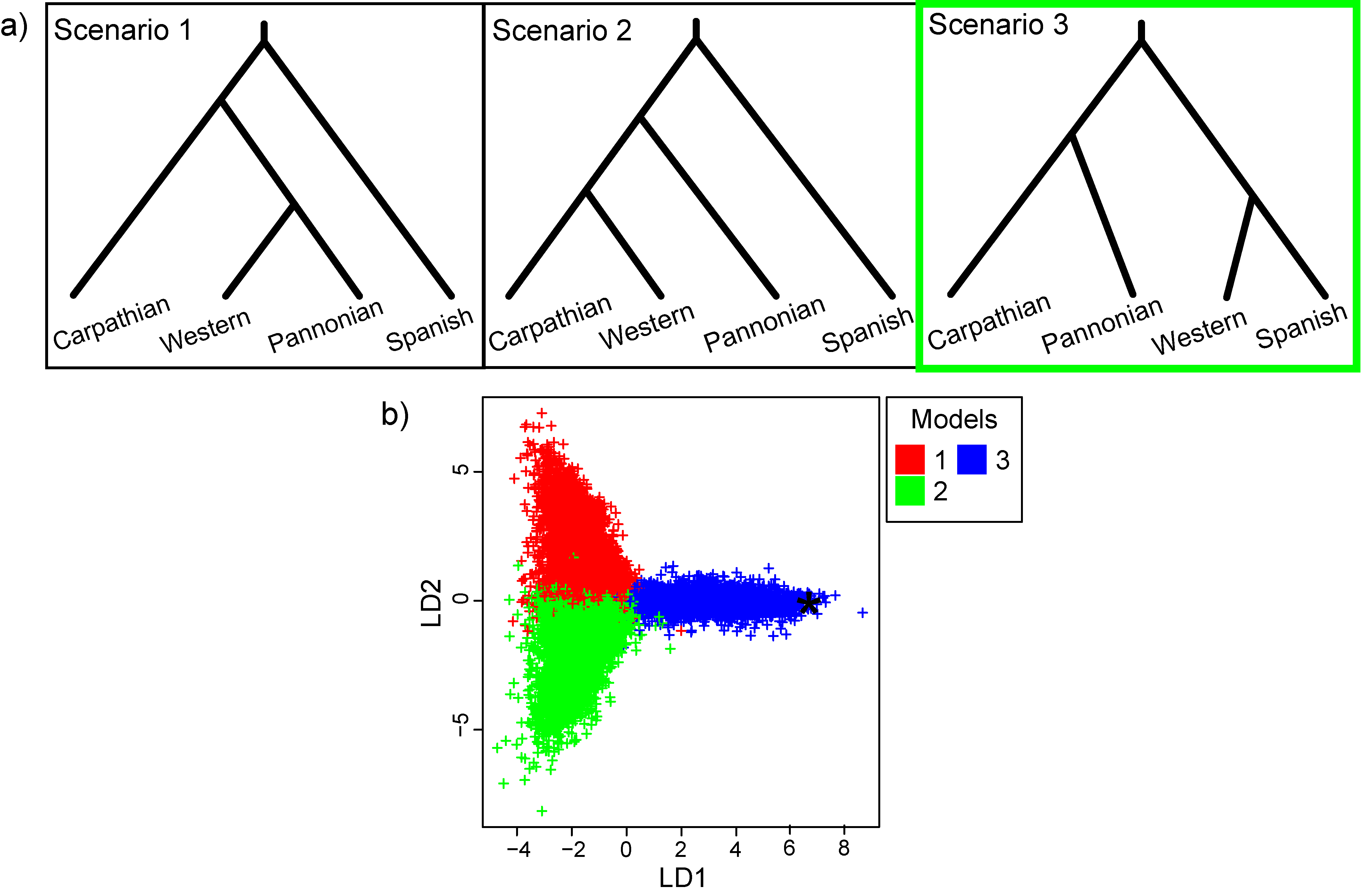

Supplement: Supplementary file 1 — Appendix S1 [file ECE3-11-8215-s001.zip › ece37652-sup-0007-FigA7.tif]

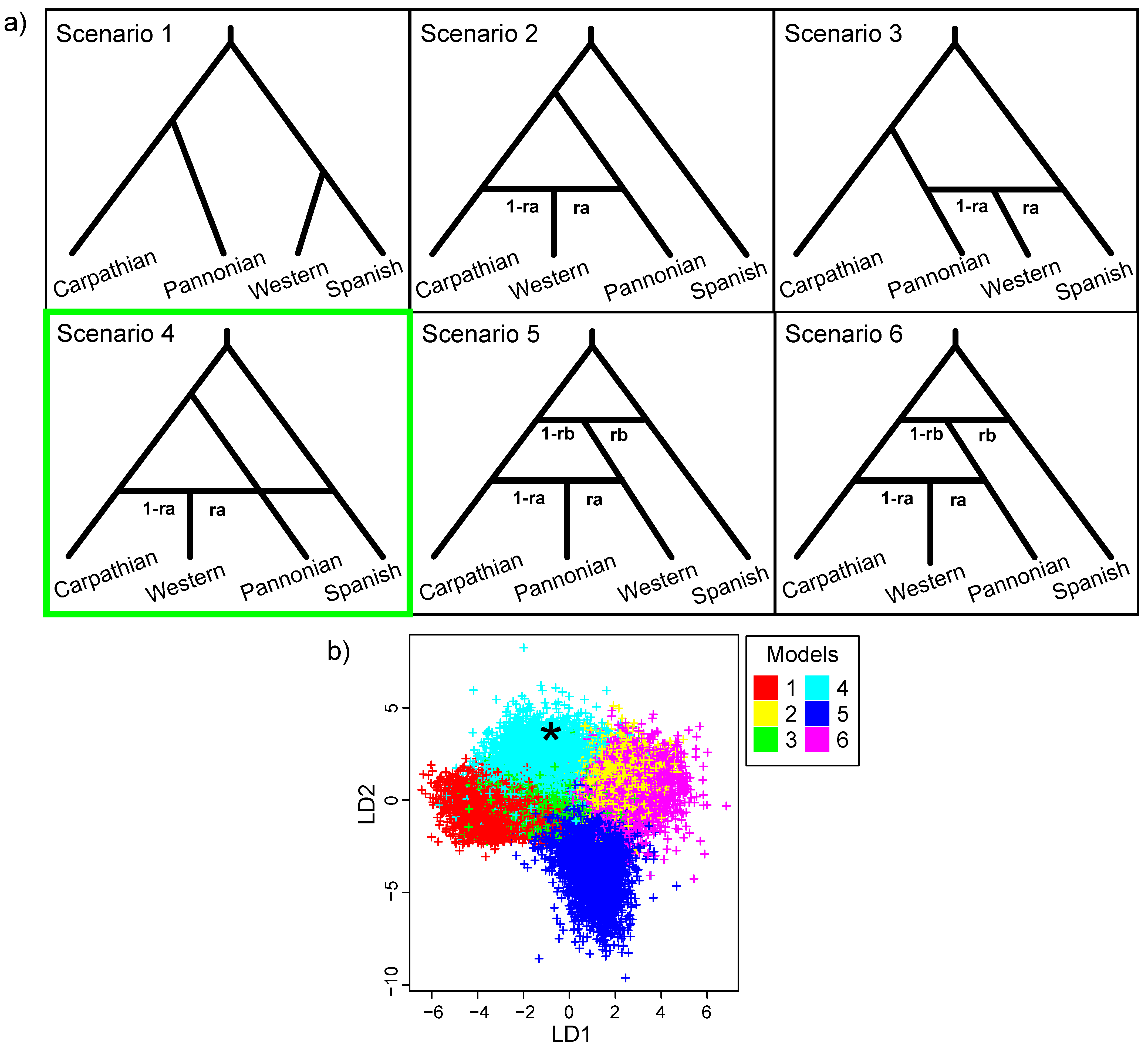

Supplement: Supplementary file 1 — Appendix S1 [file ECE3-11-8215-s001.zip › ece37652-sup-0008-FigA8.tif]
